# Pre-clinical development as microbicide of zinc tetra-ascorbo-camphorate, a novel terpenoid derivative: Potent in vitro inhibitory activity against both R5- and X4-tropic HIV-1 strains without significant in vivo mucosal toxicity

**DOI:** 10.1186/1742-6405-5-10

**Published:** 2008-06-03

**Authors:** Héla Saïdi, Mohammad-Ali Jenabian, Bernard Gombert, Charlotte Charpentier, Aurèle Mannarini, Laurent Bélec

**Affiliations:** 1 Laboratoire de Virologie, Hôpital Européen Georges Pompidou, and Université Paris Descartes (Paris V), Paris, France; 2 MGB Pharma, Nîmes, France

## Abstract

**Background:**

Terpenoid derivatives originating from many plants species, are interesting compounds with numerous biological effects, such as anti-HIV-1 activity. The zinc tetra-ascorbo-camphorate complex (or "C14"), a new monoterpenoid derivative was evaluated in vitro for its anti-HIV-1 activity on both R5- and X4-HIV-1 infection of primary target cells (macrophages, dendritic cells and T cells) and on HIV-1 transfer from dendritic cells to T cells.

**Results:**

The toxicity study was carried out in vitro and also with the New Zealand White rabbit vaginal irritation model. C14 was found to be no cytotoxic at high concentrations (CC50 > 10 μM) and showed to be a potential HIV-1 inhibitor of infection of all the primary cells tested (EC50 = 1 μM). No significant changes could be observed in cervicovaginal tissue of rabbit exposed during 10 consecutive days to formulations containing up to 20 μM of C14.

**Conclusion:**

Overall, these preclinical studies suggest that zinc tetra-ascorbo-camphorate derivative is suitable for further testing as a candidate microbicide to prevent male-to-female heterosexual acquisition of HIV-1.

## Background

Sexual transmission of HIV-1 is predominant worldwide, and male-to-female transmission during heterosexueal intercourse is the major way of HIV-1 acquisition in exposed women, especially in developing countries [1]. Interventions aimed to provide significant changes in sexual behaviour and increased frequency of barrier methods (male and female condoms) use have not proven their efficacy to decrease the HIV-1 epidemic in developing countries [2]. Therefore, new methods of prevention that can be controlled by women them-self, such as microbicide formulations, are becoming urgently needed. Microbicides may theoretically target the incoming virus at several steps of molecular events driving viral entry and/or viral replication. Unlike condoms, they will not create a physical barrier to intimate contact, nor will they necessarily be contraceptive. The fact that their use will be controlled by women obviously constitutes a very significant advantage.

Natural products, of which structural diversity is so broad, are convenient sources for the effective discovery of anti-HIV-1 agents with expected lack of cell toxicity [3-5]. Of these, terpenes, isolated from medicinal plants, have gained much interest due to their significant anti-HIV-1 activities along with their structural diversity. Betulinic alcohol (BA) is a pentacyclic triterpene alcohol with a lupane skeleton. BA is particularly promising because it is well characterized and can be purified in relatively large amounts[5,6]. Common structural features of the lupane skeleton are its five-membered ring and isopropylidene and it is found predominantly in bushes and trees forming the principal extractive of the bark of birch trees. BA possesses a wide spectrum of biological and pharmacological activities, such as antimalarial and anti-inflammatory activities[7]. BA and its derivatives have demonstrated high anti-HIV-1 activity and cytotoxicity against a variety of tumor cell lines comparable to some clinically used drugs [8]. Two classes of chemically modified BA derivatives are reported to inhibit HIV-1 replication at nanomolar concentrations, such as PA-457 (class I) and IC9564 (class II) [7]. Although both classes of BA derivatives shared the same betulinic acid core, they exhibit very different modes of anti-HIV-1 action [9]. Previous studies suggested that the molecular mechanism of action for both classes of BA derivatives were quite unique in comparison with currently known anti-HIV-1 drugs that target HIV-1 reverse transcriptase or protease [5]. Overall, based on their site of action, anti-HIV-1 terpenes could be classified into five groups: 1) entry inhibitors, 2) reverse transcriptase inhibitors, 3) protease inhibitors, 4) virus maturation inhibitors that do not inhibit HIV-1 protease and 5) unknown mechanism of action [10]. Notably, these terpenoid derivates are non-toxic up to 500 mg/kg body weight in mice[6].

The purpose of the present study was to evaluate the first steps of preclinical development of zinc tetra-ascorbo-camphorate (named as "C14"), a novel terpenoid derivative, as potential microbicide molecule. We herein report that this compound inhibited *in vitro *efficiently the infection of macrophages, dendritic cells (DC) and T cells. Standardized animal model was used to examine the safety and toxicity profiles of C14 derivative. Importantly, antiviral concentrations of C14 derivative did not result in detectable levels of inflammation or toxicity *in vivo*. Our observations strongly support that microbicide formulation containing zinc tetra-ascorbo-camphorate may represent a powerful candidate microbicide for the prevention of male-to-female HIV-1 heterosexual transmission.

## Materials and methods

### Zinc tetra-ascorbo-camphorate derivative

The zinc tetra-ascorbo-camphorate derivative of formula 4(C6H6O6)Zn(C10H14O4) contains a pentacyclic ring system obtained from a terpene of generic formula (C5H8)n and 4 ascorbic acids stably linked to an unique Zn metal. The batch used in present experiment was named as "C14". C14 was synthesized according to the following steps: 1) Preparation of a solution of organic terpenoid acid using a mixture of pure water and alcohol; 2) Reaction of the latter solution with zinc metal salt providing a new terpenoid compound associated with the metal; 3) Separation, purification and lyophilization of the resulting metallic compound; 4) Reaction of the resulting product with ascorbic acid in aqueous solution, and formation of zinc ascorbo-camphorate; 5) Separation, purification and lyophilization of the resulting product to obtain the pure final derivative in the form of a powder soluble in water. Synthesis of zinc tetra-ascorbo-camphorate derivative is around 10 cents of euro per g.

### Reagents

RPMI 1640 (with L-glutamine) and penicillin/streptomycin were provided from Cambrex, Biosciences, Verviers, Belgium and Invitrogen, Cergy-pontoise, France, respectively. Medium of separation for lymphocytes (MSL) and fetal calf serum (FCS) were from PAA Laboratories GmbH (les Mureaux, France), and Eurobio (Les Ulis, France), respectively. Human recombinant macrophage-colony stimulating factor (rhM-CSF), granulocyte-colony stimulating factor (rhM-CSF), interleukin-4 (rhIL-4) and interleukin-2 (rhIL-2) were obtained from Peprotech (Rocky Hill, NJ). Phytohemagglutinin-P (PHA) was from Sigma-Aldrich (St Louis, MO). T-20, Fusion Inhibitor (DAIDS, free N and C terminal amino acids) was obtained from the AIDS Reagent Program, Division of AIDS, NIAID, NIH. Human polyclonal anti-gp160 antibodies were purified by immunoaffinity from pooled sera of HIV-1 seropositive individuals [11].

### Antibodies

Anti-CD4 mAb (PE-CD4, RPA-T4), anti-CCR5 (PE-CCR5, 2D7), anti-CXCR4 (PE-CXCR4, 12G5), anti-HLA-DR (FITC-HLA-DR, TU-36), anti-CD14 (PE-CD14, M5E2), anti-CD16 (FITC-CD16, 3G8) and anti-DC-SIGN (PE-DC-SIGN, DCN46) mAbs were obtained from BD Pharmingen.

### Virus stocks

Primary X4-HIV-1_NDK _was grown in peripheral blood lymphocytes (PBL) of healthy donors stimulated with PHA and rhIL-2. R5-HIV-1_Ba-L _was amplified in monocyte-derived macrophages of healthy donors. Viral stock produced was clarified by centrifugation prior to HIV-1 p24 concentration and TCID_50 _determination: 1 ng of p24 antigen corresponding to 1000 TCID50 [11].

Tropism of viruses was determined using U87 cells transfected with DNA encoding for human CD4 and CCR5 or CXCR4 (NIH AIDS research and Reference Reagent Program provided by Dr. E. Menue, Institut Pasteur, Paris). The number of viral particles was assessed by the real time RT-PCR. Briefly, RNA were isolated from HIV-infected cells on a silica column system according to the manufacturer's recommendations (Qiagen DNA or RNA minikit, AG, Basel, Switzerland). HIV-1 RNA quantification was carried out by RT-PCR using primers (forward: 5'-GGCGCCACTGCTAGAGATTTT-3'; reverse: (5'-GCCTCAATAAAGCTTGCCTTGA-3') and exonuclease probe (5'-FAM-AAGTAGTGTGTGCCCGTCTGTTRTKTGACT-TAMRA-3') designed to amplify a fragment in the long terminal repeat (LTR) gene. Reverse transcription and amplification were achieved in a one step RT-PCR using the LightCycler-RNA master hybridization probes kit (Roche Diagnostics Corporation, Mannheim, Germany), as previously described [12]. A standard graph of the *Cp *values was obtained from serial dilutions (10^6 ^to 10 copies per assay) of the HIV-1 subtype A strain. Similar concentrations (expressed in copies/ml) of HIV-1_Ba-L _and HIV-1_NDK _solutions stocks were used.

### *In vitro *differentiation of monocyte-derived macrophages (MDM) and monocyte-derived dendritic cells (MDDC)

PBMC were isolated from buffy coats of healthy adult donors by Ficoll density gradient centrifugation on MSL, as previously described [13]. The percentage of monocytes was determined by flow cytometry using forward scatter and side scatter properties (FSC/SSC). PBMC were re-suspended in RPMI 1640 medium supplemented with glutamine, penicillin (100 IU/ml) and streptomycin (100 μg/ml). Cells were seeded into 24 well-plates (Costar, Cambridge, MA) at the concentration 1 × 10^6 ^adherent cells/ml and incubated at 37°C for 45 minutes. Nonadherent cells were removed by 4 washes. Adherent monocytes were incubated in RPMI medium with 10% FCS, glutamine, and antibiotics in the presence of 10 ng/ml rhM-CSF (10 ng/ml) to differentiate to macrophages. The relative concentration of rhM-CSF improve cell viability and maintained a neutral environment with respect to activation marker quantitative expression (HLA-DR, CD14, CD16), which remained similar to that of MDM cultured in medium alone. After six days of culture, adherent cells corresponding to the macrophages-enriched fraction were harvested, washed, and used for subsequent experiments [14]. MDDC are generated from monocytes in the presence of rhGM-CSF (10 ng/ml) in combination with rhIL-4 (10 ng/ml). Following six days, MDDC are semi-adherent cells and expressed high levels of DC-SIGN but not monocytes/macrophages markers such as CD14 and CD16. The medium, including all supplements, was replaced the third day of differentiation. Flow cytometry analysis (CellQuest software) demonstrated that macrophages and DCs were more than 90% pure.

### Purification of autologous T lymphocytes

T cells were subsequently prepared from the monocyte-depleted cell fraction. Peripheral blood lymphocytes (PBL) were cultured for 48 hours in fresh medium supplemented with PHA (2.5 μg/ml) and rhIL-2 (1 μg/ml). PBL were then washed and cultured in growth medium containing rhIL-2 (1 μg/ml) for 24 hours [14].

### Phenotypic characterization of MDM or MDDC

Cell surface antigens were analyzed by FACSCalibur (Becton Dickinson, NJ, USA) using monoclonal antibodies (mAbs) conjugated with either fluorescein isothiocyanate (FITC) or phyco-erithryn (PE). Following incubation with different mAbs for 30 min at 4°C, cells were washed with PBS containing azide (0.01%), BSA (0.2%) and fixed using 1% formaldehyde PBS buffer.

### Inhibition of infection of MDM, MDDC or T cells [13,14]

Cells were washed 2 times after 6 days of differentiation and seeded into 96-well culture plates (5 × 10^5 ^cells/well). HIV-1 (1 ng p24 antigen/ml) and increasing concentrations of molecules were added on cells and incubated for 3 hours at 37°C in a 5% CO_2 _atmosphere. Each sample was performed in triplicate. After 4 washes to remove exceeding virus, cells were cultured for 3 days. The amounts of virus replication were monitored by HIV-1 p24 antigen ELISA, so carried out 3 days after exogenous addition of C14. In this last case, supernatants were harvested and viruses produced were lysed by incubation for 45 minutes at 37°C with 1% Triton X-100.

### Extraction and quantification of HIV-1 DNA

Genomic DNA was isolated from HIV-infected macrophages by using extraction protocol on a silica column system according to the manufacturer's instructions (Qiagen DNA minikit, AG, Basel, Switzerland). HIV-1 DNA was quantified by using 5' nuclease assay in the LTR gene and carried out on the LightCycler instrument (Roche Applied Science), with using the sense primer NEC152 (GCCTCAATAAAGCTTGCCTTGA) and the reverse primer NEC131 (GGCGCCA CTGCTAGAGATTTT) in the presence of a dually (FAM and TAMRA) labelled NEC LTR probe (AAGTAGTGTGTGCCCGTCTGTTRTKTGACT) (Eurogentec SA, Seraing, Belgium). The LC-PCR master mix contained 1 × Fast-Start Taq DNA polymerase reaction buffer (Roche Applied Science), 3 mM MgCl_2_, 0.3 μM of each primer and probe. Cycling conditions were as follows: initial denaturation/FastStart Taq DNA polymerase activation at 95°C/10 minutes, 45 cycles of denaturation at 95°C/10 seconds, annealing and extension at 60°C/30 seconds with a ramp of 5°C/seconds. The first PCR cycle allowing fluorescence detection permitted to quantify HIV-1 DNA by reference to a standard curve (dilutions of 8E5 cell DNA). All reactions were performed in triplicate and tested in the same assay. The level of albumin DNA copies in the cell pellet was used as endogenous reference to normalize the variations in cells number, as previously described [15]. The normalized value of cell-associated HIV-1 DNA loads corresponding to the ratio [(HIV-1 copy number/albumin copy number) × 2 × 10^6^], was finally expressed as the number of HIV-1 DNA copies per 10^6 ^cells.

### Inhibition of MDDC-mediated infection of autologous T cells [14]

To assess the transmission of HIV-1 from MDDC to autologous T-cells, MDDC were incubated into 96-well culture plates (10^5 ^cells/well) and infected with HIV-1 (1 ng p24) in the presence of increasing concentrations of molecules for 3 hours at 37°C in a 5% CO_2 _atmosphere. Cells were washed four times and autologous stimulated T cells were added onto infected MDDC at a MDDC/T-cell ratio of 1/5 for 6 days. Each sample was performed in triplicate. Culture supernatants were harvested every 3 days and fresh medium was added. Supernatants were inactivated with 1% Triton X-100. The viral production by T lymphocytes was evaluated the sixth day of the co-culture by measurement of HIV-1 p24 antigen in supernatants using capture ELISA.

### Cytotoxicity assay

The cytotoxicity of the C14 derivative against primary cells (MDDC, T cells and MDM) was analysed using the MTT (3-[4,5-dimethylthiazol-2-yl]-2,5-diphenyl tetrazolium bromide) assay (Sigma-Aldrich), as previously described [14]. Briefly, cells were seeded onto 96-well plates at a density of 2 × 10^5 ^cells/well and incubated for 24 hours at 37°C prior to drug exposure. On the day of treatment, culture medium was carefully aspirated from the wells and replaced with fresh medium containing serial concentrations of C14 derivatives. Triplicate wells were used for each treatment. The cells were incubated with the various compounds for 24 hours at 37°C in a humidified 5% CO_2 _atmosphere. To each well, 20 μl of MTT (0.5 mg/ml final concentration) was added and the plates were incubated at 37°C for 4 hours to allow MTT to form formazan crystals by reacting with metabolically active cells. The formazan crystals were solubilized 30 minutes at 37°C in a solution containing 10% sodium dodecyl sulphate in 0.01 M HCl. The absorbance of each well was measured in a microtitre reader at 490 nm. To translate the OD_490 _values into the number of live cells in each well, the OD_490 _values were compared with those of standard OD_490 _versus cell number curves generated for each cell type. The survival index was calculated using the formula:

Survival index = live cell number (test)/live cell number (control)

### Confocal microscopy

Monocytes or PBL (10E5 cells) were adsorbed on a microscopy-adapted slide for 6 days. MDDC, MDM or PBL were infected in the presence of C14 diluted at 10 μM for 3 hours. Cells were then washed and incubated with or without polyclonal antibodies anti-gp160 (50 μg/ml) at 4°C for 30 minutes. Cells were washed with PBS 0.01% azide 0.5% BSA, and were labelled with polyclonal mouse anti-human IgG-FITC (Jackson ImmunoResearch Laboratories, West Grove, PA, USA) and then fixed with 1% paraformaldehyde. The coverslides were mounted in Mowiol (Sigma-Aldrich). The observations were made by sequential acquisition with a Zeiss LSM510 System, mounted on an Axiovert 100 M optical microscope (Carl Zeiss AG, Oberkochen, Germany), using a planapochromat ×63, 1.4 numerical aperture oil immersion objective. Optical sections were acquired, each one with an image resolution of 512 × 512 pixels.

### New Zealand White rabbit vaginal irritation study

All procedures for the rabbit irritation study were conducted in referring to French authorities («ISO 10993 standard, version 2002: Biological Evaluation of Medical Devices, Part 10: Tests for irritation and sensitization»), and this part of the study was performed by the Biomatech company (Chasse-sur-Rhone, France) which is certified according to the European qualification ISO 17025.

Nine nulliparous and nonpregnant female New Zealand White rabbits were used to determine potential irritation effects following vaginal application of two C14 formulations. All animals were acclimated for 5 days prior to the experiment. The animals were categorized into 3 treatment groups, including three rabbits treated with low dose of C14 (C14 diluted 2000 times in PBS, 1 μM; C14-LD), three with high dose of C14 (C14 diluted 100 times in PBS, 20 μM; C14-HD), and three PBS-treated animals. The animals received vaginally 1 ml of C14 or PBS per day for 10 consecutive days. The animals' body weights were measured daily; and clinical observations were recorded, including swollen vulva areas, blood-stained urine, and soft stools. On day 10, all animals were euthanized by intravenous injection of sodium pentobarbital, in accordance with the guidelines of the American Veterinary Medical Association Panel on Euthanasia. The vaginal tracts were surgically excised and parts of the upper (cervicovagina), middle (midvagina), and lower (urovagina) areas of each vagina were fixed with formalin and paraffin embedded by standard histological examination. To assess gross tissue morphology, sections were stained with hematoxylin and eosin. A vaginal irritation grading system with scores from 0 (normal parameter or absent adverse effects) to 4 (most severe adverse findings) was used to score each formulation for epithelial integrity, epithelial vascular congestion, leukocyte infiltration, and edema. Composite average scores of 1 to 4 receive a vaginal irritation rating of "minimal," scores of 5 to 8 receive a vaginal irritation rating of "mild," scores of 9 to 11 receive a vaginal irritation rating of "borderline," and scores of 12 to 16, receive a vaginal irritation rating of "unacceptable". Formulations with vaginal irritation ratings between 1 and 8 are considered acceptable for vaginal application [16].

### Statistical analysis

Statistical significance of the treated group mean with that of control group was analyzed by a 1 way-analysis of variance, followed by Dunnett's multiple comparison test using GraphPad Prism version 3.0 software (San Diego, CA). Differences were considered statistically significant if p < 0.05.

## Results

### High concentrations of C14 are not toxic *in vitro*

**Figure 1:**
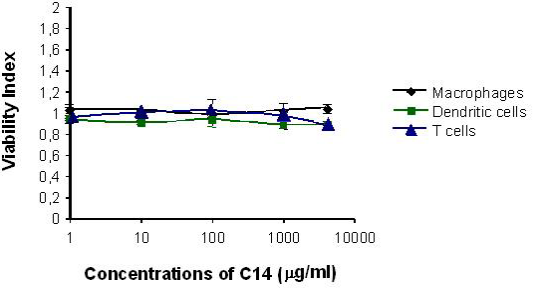
**Evaluation of C14 toxicity on primary cells.** Monocyte-derived macrophages, monocyte-derived dendritic cells and T cells were cultured with increased concentrations of C14 (ranged from 1 to 12 μM) for 24 hours. After washing, culture viability was determined by using the MTT-cytotoxicity assay according to the manufacturer's instructions. The values given are the mean viability ± 1 standard deviation of primary cells, expressed in percentage. Means are representative of 3 independent experiments and assays were performed in duplicates.

High concentrations of C14 derivate may be needed to produce an effective microbicide formulation. Therefore, the intrinsic toxicity of C14 derivate concentrations up to 5 μM was evaluated by using a colorimetric cell viability assay. MDM, MDDC and T cells were exposed to serial concentrations of each C14 derivate ranging from 1 to 12 μM for 24 hours. The viability index, or the fraction of viable cells following microbicide treatment relative to the fraction of viable mock-exposed cells, was calculated. Cells treated by a solution of PBS-azide 0.1% were used as a positive control for toxicity (data not shown). As shown in Figure 1, C14 demonstrated viability indices of 0.9 to 1.1 at all concentrations tested, which indicated that it was non-toxic.

### C14 inhibits HIV-1 infection of primary cells

Predominant HIV-1 target cells at mucosal level include CD4 T lymphocytes, submucosal macrophages, intraepithelial and submucosal DCs [17,18]. The amounts of virus replication were monitored by HIV-1 p24 antigen ELISA, so carried out 3 days after exogenous addition of C14. Indeed, successful transfer of virus across epithelial barriers would result in HIV-1 capture by DC and subsequent transmission to nearby macrophages and CD4 T cells or dissemination to draining lymph nodes [17]. We first investigated the effect of C14 on HIV-1 infection of macrophages, DC and T cells. Therefore, HIV-1 sensitive cells were incubated with R5-HIV-1_Ba-L _or X4-HIV-1_NDK _in the presence of increasing non-toxic doses of C14 (Table 1). C14 inhibited efficiently the infection of all cell type tested and whatever the viral strain tested. Enfuviritide (T-20) interfere with entry of the HIV-1 virus into cells by blocking the structural changes necessary for virus to fuse with CD4+ cell membrane and inhibiting then fusion of viral and cellular membranes, which served as positive control in our tests evaluating the inhibitory activity of C14. Conversely to T-20, much lower doses of C14 were needed to inhibit more efficiently the infection of macrophages and T cells by X4 virus.

**Table 1:** Toxicity and antiviral activity of zinc tetra-ascorbo-camphorate derivative ("C14") on macrophages, dendritic cells and peripheral blood lymphocytes by using the primary X4-tropic HIV-1_NDK _and R5-tropic HIV-1_Ba-L_.

**Antiviral molecules**	**CC50**^a^			**IC50**^b^					
	**MΦ**	**DC**	**T cells**	MΦ*		DC*		T cells*	
	
				**HIV-1**_Ba-L_	**HIV-1**_NDK_	**HIV-1**_Ba-L_	**HIV-1**_NDK_	**HIV-1**_Ba-L_	**HIV-1**_NDK_
**C14**	>10	>10	>10	1.3 ± 01	0.02 ± 0.0	1.3 ± 0.1	1.8 ± 0.1	0.8 ± 0.0	0.7 ± 0.1

**Enfuviritid (T20)**	>10	>10	>10	0.08 ± 0.1	8 ± 0.5	0.3 ± 0.0	0.8 ± 0.3	0.4 ± 0.2	6.7 ± 0.2

*Mean ± 1 standard deviation

^a^Terpenoid derivative C14 concentration (μM) that causes 50% cytotoxicity (CC50) on primary cells (MΦ, DC, T cells)

^b^Terpenoid derivative C14 concentration (μM) that induces 50% infection inhibition (IC50) on primary cells (MΦ, DC, PBL)by primary X4-HIV-1_NDK_or R5-HIV-1_Ba-L_, expressed as mean ± 1 standard deviation MΦ: Macrophages; DC: Dendritic cells

To study the antiviral activity of C14 on HIV-1 transfer from DC to T cells, DC were pre-treated with C14 followed by addition of cell-free HIV-1. After infection, cultures were washed and co-cultured with autologous CD4 T cells, without C14, and half of the culture supernatant was refreshed twice weekly with culture medium without compound. Culture supernatants were harvested after 7 of culture for measurement of HIV p24 antigen. At 1 μM, C14 inhibited about 95% both the transfer of R5- and X4-tropic HIV-1 (data not shown).

### HIV-1 DNA content of C14-treated cells is very low

**Figure 2:**
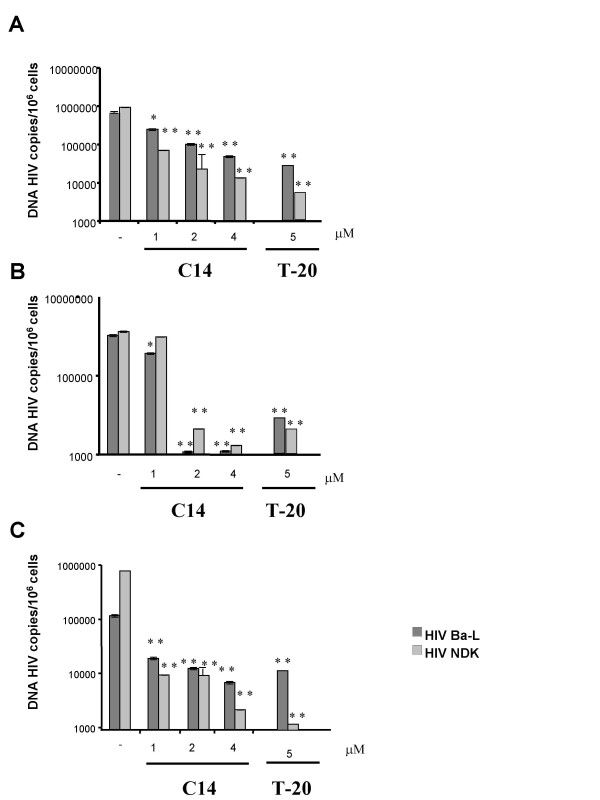
**Evaluation of C14 inhibitory activity on HIV-1 DNA content into primary cells. **Monocyte-derived macrophages (A), monocyte-derived dendritic cells (B) and T cells (C) were incubated with R5 or X4 viruses in the presence of increasing concentrations of C14 or an unique dose of T 20 (5 μM) for 3 hours at 37°C. Cells were then washed and cultured in fresh medium for 3 days. DNA was extracted and the viral DNA was quantified by real time PCR. Means are representative of 3 independent experiments and assays were performed in triplicates. *, *p *≤ 0.05; **, *p *≤ 0.01 between untreated and treated cells.

*In vivo*, macrophages, DC and CD4 T cells are described to be major reservoir of HIV-1 [19]. Cells were incubated with R5-HIV-1_Ba-L _or X4-HIV-1_NDK _in the presence of increasing doses of C14. The HIV-1 DNA content was quantified precisely with a optimised and randomised method of real time PCR. As depicted in Figure 2, we observed a reduction between 2–3 log of the HIV-1 DNA content in all primary cells and whatever the viral strain used. FACSCalibur analysis revealed that the cell surface expressions of CD4, CXCR4 and CCR5 on the primary cells we used were not altered by their treatment with the C14 compound (data not shown). Thus, the observed reduction of HIV-1 proviral DNA levels within C14-treated cells was not due to the effect of C14 on cellular expression of HIV-1 receptors and co-receptors, but rather the direct effect of C14 on the integrity of viral particles or the inhibition of viral entry.

**Figure 3:**
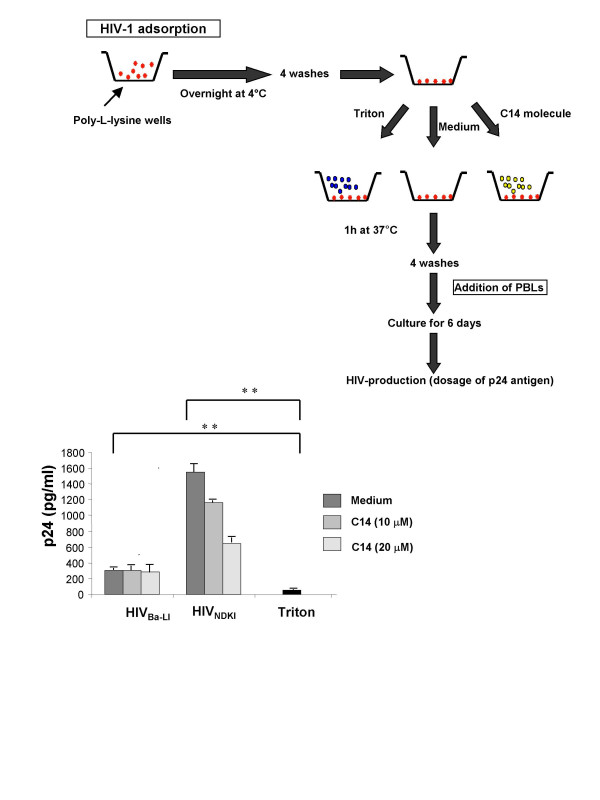
**Evaluation of C14 capability to limit the infectiousness of viral particles.** HIV-1 was adsorbed on poly-L-lysin pre-coated wells (Greiner Bio-One) at 4°C overnight and further incubated with C14 for 1 h. In positive and negative control wells, 1% Triton X-100 and medium were added, respectively. After four washes, activated peripheral blood lymphocytes were incubated with C14 or triton-treated or untreated HIV-1 particles. After 6 days, viral production was assessed by p24 Ag capture ELISA. Means are representative of 3 independent experiments were performed in triplicates. **, *p *≤ 0.01.

In a first approach, we determined whether C14 could disrupt HIV-1 particles leading to an inactivation of HIV-1 particles infectiousness. HIV-1 was thus adsorbed on poly-L-lysin pre-coated wells and further incubated with or without C14. To assess the infectiousness of these C14 treated-HIV-1 particles, activated PBL, well known producers of high levels of HIV-1 [20], were incubated with C14-treated or untreated HIV-1 particles. The levels of R5-tropic HIV-1_Ba-L _production by activated PBL were similar in wells containing cells co-cultured with virus treated or untreated with C14 (Figure 3). However, at high concentrations (20 μM), C14 was able to disrupt 58 ± 2% of X4-tropic HIV-1 and not R5-tropic HIV-1 particles. As expected, no viral production was detected in control wells containing triton X100-treated HIV-1 and activated PBL (negative control).

**Figure 4:**
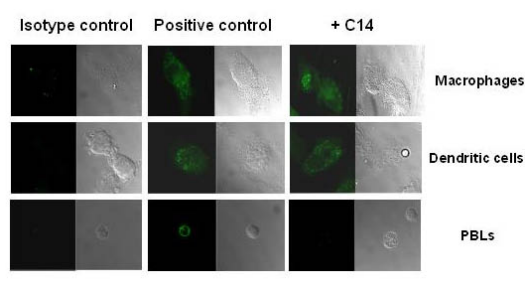
**Evaluation of C14 efficiency to inhibit limit the entry of HIV-1 into primary cells. **Monocyte-derived macrophages, monocyte-derived dendritic cells and T cells were incubated with R5 viruses in the presence of increasing concentrations of C14 for 3 hours at 37°C. Cells were then washed and incubated with polyclonal human anti-gp160 antibodies. The staining was revealed with FITC-conjugated mouse anti-human IgG mAbs. Cells were then analysed by confocal microscopy. The experiment was performed 3 times with cells from three different donors. 30 cells were at least analyzed for each donor.

We determined further whether C14 could inhibit the entry of viruses into primary macrophages, DC and T cells (Figure 4). Cells were incubated with R5-tropic HIV-1_Ba-L _in the presence of 1 μM of C14 for 3 hours. After several washes, cells were incubated with polyclonal antibodies anti-gp160 purified from HIV-infected patients and then with anti-human IgG-FITC mouse antibodies. As observed by immunofluorescence confocal laser microscopy, C14 inhibited the entry of viruses only into T cells, unlike in macrophages and DCs.

Taken together, these data strongly suggest that C14 could alter the infectiousness of viruses, inhibiting the entry of viruses into T cells and interfering with the HIV-1 reverse transcriptase activity.

### C14 derivative does not cause significant cervicovaginal inflammation or general toxic effects

**Figure 5:**
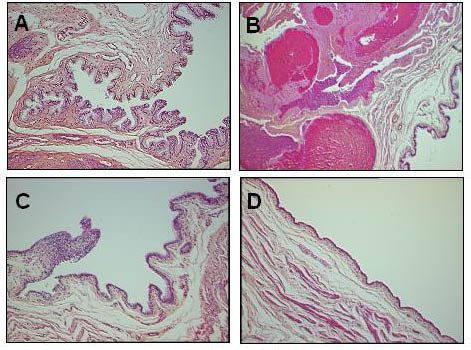
**Effects of low and high concentrations (1 and 20 μM) of C14 on cervicovaginal mucosa in the New Zealand White rabbit model.** Rabbit cervicovaginal epithelium was treated with 1 or 20 μM of C14. The vaginal tracts were surgically excised, formalin fixed, and paraffin embedded by standard histological protocols. To assess gross tissue morphology, sections were stained with hematoxylin and eosin. (A) Normal histopathological aspect of the mucosal epithelium of the rabbit #436 that was treated with C14 (1 μM) for 10 days (× 300). (B) Vascular congestion of the mucosal epithelium of the rabbit #435 that was treated with C14 (20 μM) for 10 days (× 300). (C) Vascular congestion of the mucosal epithelium of the rabbit #436 that was treated with C14 (1 μM) for 10 days (× 100). (D) Erosion of the mucosal epithelium of the rabbit #432 that was treated with C14 (20 μM) for 10 days (× 300).

The New Zealand White rabbit model was used to assess whether repeated applications of C14 derivate resulted in vaginal irritation. Animals were treated with 1 ml of PBS dilution of C14 containing either low dose (1 μM; C14-LD) or high dose (20 μM; C14-HD) of the compound. Animals treated with PBS alone were included as a reference control. These animals that did not receive compound were subjected to the same technical application protocol as the treated animals. All animals received daily intravaginal doses of C14 for 10 consecutive days. Twenty-four hours after the last application of the test articles, the vaginal tracts were excised from all animals and processed for histopathological evaluation. There was no mortality, and no vaginal discharge, erythema or edema were noted during the study in rabbits injected with the test solutions. As expected, PBS-treated animals exhibited normal tissue morphology and staining profiles. The vaginal tissues taken from animals treated with C14-LD or C14-HD showed some polymorphonuclear cell infiltration in the epithelial and sub-epithelial connective tissue. However, the infiltration observed and vascular congestion was not general and was graded as "minimal" and edema could not be observed (Table 2) (Figures 5A and 5B). The vaginal epithelium remained quite intact (Figures 5A, 5B and 5C), and in two rabbits treated with C14-HD, local minimal erosion of the mucosal surface was noted (Figure 5D). Squamous metaplasia and focal erosion were occasionally observed with a degree of leukocyte infiltration primarily in the sub-epithelial connective tissues. No necrosis or vascular thrombi were observed in any animals during this study. Standardized microscopic evaluation criteria were used to assign a composite average score for each test formulation. The means of vaginal irritation index for each of the test groups were as follows: C14-HD-treated group, 2.4 and C14-LD-treated group, 2.1; PBS-treated control, 0.2 (Table 2).

**Table 2:** Scores of epithelium irritation, leucocytes infiltration, vascular congestion and edeme, and vaginal irritation index obtained in three groups of New Zealand White rabbits traited vaginally during 10 days by low (1 μM; C14-LD) or high doses (20 μM; C14-HD) of zinc tetra-ascorbo-camphorate derivative (C14), or by PBS (negative controls).

	C14-HD*	C14-LD*	PBS*
**Epithelium (0–4)£**	1.560 ± 1.667	0.889 ± 1.270	0
**Infiltration of leucocytes (0–4)£**	0.556 ± 0.880	0.444 ± 0.520	0.222 ± 0.440
**Vascular congestion (0–4)£**	0.444 ± 1.330	0.778 ± 1.394	0
**Edeme (0–4)£**	0	0	0
**Vaginal irritation index (0–16)$**	**2.4**	**2.1**	**0.2**

* Mean ± 1 standard deviation

£ The scores for epithelium irritation, leucocytes infiltration, vascular congestion and oedeme, were calculated as the mean of the scores estimated at the cervicovagina, midvagina and urovagina of the 3 rabbits in each group. The final score is then expressed as mean ± 1 standard deviation of 9 determinations.

$ The mean vaginal irritation index corresponds to the addition of the scores of epithelium irritation, leucocytes infiltration, vascular congestion and edeme, as adapted from the norme "ISO 10993 standard, version 2002: Biological Evaluation of Medical Devices, Part 10: Tests for Irritation and Sensitization". Interpretation of the vaginal irritation index is as follows: 0: None; 1: Minimal; 2: Mild; 3: Moderate; 4: Intense. The correlations with human irritation potential are as follows: Vaginal irritation index < 8: Acceptable; 9–10: Marginal; and ≥ 11: Unacceptable, according to Eckstein and colleagues (Eckstein *et al.*, 1969).

## Discussion

In the present study, the zinc tetra-ascorbo-camphorate complex, a new monoterpenoid derivative, was evaluated in vitro for its anti-HIV-1 activity on both R5- and X4-tropic HIV-1 infection of primary target cell (macrophages, DC and T cells) and on HIV-1 transfer from DC to T cells, and for its potential toxicity for the vaginal mucosa using the normalized rabbit vaginal irritation assay. Thus, the C14 compound used in the study showed potent HIV-1 inhibitor with IC50 of 1 μM in the different primary cells, and was also able to inhibit the transfer of HIV-1 from MDDC to autologous CD4^+ ^T lymphocytes. In addition, the compound was found to be no cytotoxic at high concentrations (CC50 > 10 μM) and lack of significant inflammation and adverse changes could be observed in rabbit cervico-vaginal tissue integrity after repeated exposure during 10 days to formulations containing up to 20 μM of C14. Taken together, our preclinical studies demonstrate that the zinc tetra-ascorbo-camphorate derivative harbours potent anti-HIV-1 activity *in vitro *without a significant *in vivo *mucosal toxicity, and thus may be suitable for further steps of microbicide development, according to the guidelines proposed by the «International Working Group on Microbicide»[21].

In our assays, C14 showed a powerful anti-HIV-1 activity depending on its concentrations. At concentration less than 1 μM, C14 inhibited *in vitro *the infection of macrophages, DC and T cells that are the first cells targeted by HIV-1 *in vivo*. Interestingly, at low concentrations, C14 inhibited more than 90% of both R5- and X4-tropic HIV-1 transfer from DC to autologous T cells, a mechanism responsible of the dissemination of the virus from the mucosal site of its penetration [22,23]. At elevated concentrations (higher than 10 μM), C14 seems to disrupt the integrity of virus particles, but at non-toxic concentrations, C14 derivate inhibited HIV-1 entry only into T cells and not into macrophages and DC, and decreased dramatically DNA proviral quantity by 1 to 3 log10 into all primary cells tested, suggesting that its antiviral activity is mostly due to its capacity to inhibit the entry of HIV-1 into T cells and may limit the reverse transcription step into macrophages and DC. These findings indicate that C14 harbours potent HIV-1 entry inhibition activity and/or targets pre-integrative step of viral cycle. Further work is needed to determine precisely the molecular mechanism of action of C14.

We have showed that the C14 compound was also able to inhibit the transfer of HIV-1 from MDDC to autologous CD4 T lymphocytes. In our experimental conditions, HIV-1 was transferred from MDDC towards T cells by mechanisms *in trans *[22] and *in cis *[24]. In addition, C14 dramatically decreased the infection of DCs. Since viruses produced by DCs (excluding virions captured and transferred *in trans *by a mechanism DC-SIGN-dependent) may be efficiently transferred from DCs to T cells [24], the observed decrease of HIV-1 transfer from DCs to T cells in the presence of C14 may result from reduced efficiency of C14-treated DCs to produce viruses. We cannot however exclude in our assay that C14 might alter immune function of DCs, which in turn may lead to a decrease in HIV-1 transfer to T cells.

The cytotoxicity for host primary cells of a non-specific anti-HIV-1 compound is a major issue. Indeed, the nonoxynol-9, a non-specific surfactant, which destroys HIV-1 particles *in vitro *[25], caused lesions in the vaginal epithelium in vivo and increased the probability of being infected with HIV-1 [26]. To assess whether biological activity of C14 causes inflammation or irritation which could subsequently promote infection, we used the standardized New Zealand White rabbit vaginal irritation model. Eckstein and colleagues reported that the rabbit vaginal test is slightly more sensitive that the monkey test and more closely reflects the likely clinical condition in humans [16]. Importantly, the rabbit test is also quicker, cheaper and more easily carried out and interpreted [16]. Notably, this model system is an advised *in vivo *assay for all candidate vaginal microbicides advancing into clinical trials [21]. Conversely to the nonoxynol-9 that have vaginal toxicity with a score of histological changes in the New Zealand White rabbit of about 8 ± 3 [27], C14 showed vaginal irritation indexes within ranges indicating that this compound may be likely suitable for vaginal use in humans [28,29].

## Conclusion

In conclusion, the high anti-HIV-1 activity, and excellent safety profile and low cost production of the zinc tetra-ascorbo-camphorate complex evaluated in our preclinical study provides strong support for the advancement of C14 as a vaginal microbicide. Further studies should include validation of C14 activity in the macaque model of experimental transmission of SIVmac251 after vaginal deposition [21] and phases I and II in focussing on tolerance in women [30].

## Competing interests

The authors declare that they have no competing interests.

## Authors' contributions

HS and MAJ carried out differentiation and infection of dendritic cells, macrophages and T cells, isolation of T cells, HIV-1 transfer assays, cytotoxicity assay, confocal microscopy assay, interpretation rabbit vaginal irritation model and helped draft the manuscript. CC carried out the extraction and quantification of HIV-1 DNA and p24 ELISA. BG and AM provided C14, participated in the design of the study, and helped draft the manuscript. LB: conceived the study, participated in its design and coordination. All authors read and approved the final manuscript.
